# Is fat suppression in T1 and T2 FSE with mDixon superior to the frequency selection-based SPAIR technique in musculoskeletal tumor imaging?

**DOI:** 10.1007/s00256-019-03227-8

**Published:** 2019-06-01

**Authors:** Willemijn H. F. Huijgen, Catherina S. P. van Rijswijk, Johan L. Bloem

**Affiliations:** 1 Leiden University Medical Centre (LUMC), Albinusdreef 2, 2333 ZA Leiden, The Netherlands; 2 Radiology Department, PO Box 9600, 2300 RC Leiden, The Netherlands

**Keywords:** Magnetic resonance imaging, Dixon, SPAIR, Fat suppression, Image quality, Homogeneity

## Abstract

**Objective:**

To determine the image quality of fast spin echo (FSE) with mDixon relative to spectral attenuated inversion recovery (SPAIR) FSE sequences in musculoskeletal tumor imaging on a 1.5-T MRI system.

**Materials and methods:**

In a HIPAA-compliant prospective study, 265 patients requiring musculoskeletal tumor MRI scans were included. Patient consent was waived by the medical ethical committee. Two radiologists compared SPAIR and mDixon FSE water-only images in both T2- and T1-weighted gadolinium-enhanced (T1-Gd) sequences using a five-point scale (paired samples* t* test and visual grading characteristics curves (VGC)). Homogeneity of fat suppression, noise, contrast, several artifacts (motion, phase, edge blurring and water–fat swap) and subjective preference were evaluated.

**Results:**

Readers did not have subjective preference for either sequence in 71% and 55% (reader 1 and 2, respectively). Scores for homogeneous fat suppression were significantly (*p* < 0.01) higher for mDixon (4.88 in T2 and 4.87 in T1-Gd) than for SPAIR (4.31 for T2 and 4.21 for T1-Gd). All VGC curves for homogeneity demonstrated preference for mDixon. In 57 individual mDixon cases, fat-suppression homogeneity was strikingly better (≥ 2 points higher), namely in areas with field heterogeneity. Average noise and contrast scores were slightly higher for mDixon, as were motion artifact scores for SPAIR (< 0.5 points difference).

**Conclusions:**

mDixon fat suppression was significantly more homogeneous than SPAIR on both T2 and T1-Gd FSE images in musculoskeletal tumor protocols. In areas of field inhomogeneity, mDixon outperforms SPAIR. SPAIR had slightly less motion artifacts than mDixon.

## Introduction

Fat suppression in musculoskeletal oncology magnetic resonance imaging (MRI) is used for improving lesion conspicuity and lesion characterization. Since the introduction of turbo or fast spin echo sequences (FSE), fat suppression has become indispensable because decreased J-coupling causes high signal intensity of fat in these sequences [1]. Some radiologists, however, prefer using T2-weighted images without fat suppression because the signal-to-noise ratio (SNR) is higher, depiction of anatomy is easier, and problems with inhomogeneous fat suppression do not exist.

Several methods of fat suppression have been developed and implemented over the years, each with their own advantages and limitations. These techniques are often based on chemical shift, non-selective inversion pulses, and hybrid techniques. In the 1980s, Dixon introduced a chemical shift method based on phase shift secondary to water–fat resonance frequency differences. The method allowed the separation of water and fat signals to be postponed to the image post-processing phase, and required only a single data acquisition sequence with multiple echo times [2]. Relative to other fat-suppression techniques, the classic Dixon techniques have long acquisition times and high sensitivity to B_0_ heterogeneity. However, because the separation of fat and water takes place during image reconstruction, the main advantages are independency to field strength and decreased sensitivity to B_1_ field heterogeneity [3]. Despite several improvements in hardware and software including multiple-point sequences, phase correction, and parallel imaging [4, 5], the still relatively long acquisition times made this sequence unpopular in routine clinical practice. A more recent modified Dixon (mDixon FSE) sequence uses two-point Dixon with flexible echo times rather than fixed in- and opposed phase echoes [3, 6]. Decreased echo times and lowered pixel bandwidth result in more efficient data acquisition and higher SNR. This allows reduction of acquisition times and reduces sensitivity to B0 heterogeneity compared to classic two-point Dixon, while maintaining the advantages of accurate water and fat separation in reconstructed images and reduced dependency on high field strength. Several studies have described superior image quality of various modern Dixon techniques compared to conventional fat-suppression methods in MSK imaging [7–14]. These studies mostly assessed image quality in specific anatomical sites.

Our aim was to determine the image quality of water-only mDixon FSE in musculoskeletal tumor imaging compared to frequency selective fat-suppressed-based spectral attenuated inversion recovery (SPAIR) FSE sequences in T2- and T1-weighed gadolinium-chelate enhanced (T1-Gd) images, in a specific tumor protocol on a 1.5-Tesla MRI system.

## Materials and methods

### Patients

In this prospective study, during a 2-year period (2015-2016), all consecutive patients who required a musculoskeletal tumor scan on the same 1.5-T MRI scanner were included. Because all studies were clinically indicated, patient consent was waived by the local medical ethical committee.

All untreated new patients and patients being treated or under surveillance were eligible. Indications were diagnosis, staging, therapy monitoring or detection of recurrence. From 330 eligible patients, 66 were excluded because of technical protocol violations such as use of tailored pulse sequence improvements. During the first year (129 patients), a T1-Gd mDixon sequence was added to the standard protocol. In the second year (135 patients), a T2-weighted mDixon sequence was used instead. Out of 264 patients, 132 were male. Mean age for males was 49.7 (range, 11–83) years and for females was 48.2 (range, 12–88) years. Mean age for all patients was 48.9 (range, 11–88) years.

### Protocol

All scans were performed using the same 1.5-T MRI system (Philips Ingenia, Release 5.3.0.3, Best, The Netherlands). Surface coils and scanning parameters depended on the body part imaged: for the shoulder, mediastinum, trunk, and pelvis the 32 channel torso surface coil was used, for the knee the 16 channel knee coil and for the other extremities the eight-channel small extremity coil. All elements of these coils were active during scanning. Standard musculoskeletal tumor protocol included SPAIR fat suppression for both axial T2- and multiplanar T1-weighted FSE Gd-chelate (0.2 cc/kg of Gadoterate Meglumine, Dotarem, Guerbet, Cedex, France) enhanced sequences. To this protocol, either a T2-weighted mDixon FSE or Gd-chelate enhanced T1-weighted mDixon FSE sequence was added. The mDixon and SPAIR sequences were performed in the same session, on the same patient, and the surface coil, scanning plane, slice thickness, and resolution were the same for both sequences. Between patients, however, these parameters varied, depending on the body part that was imaged. Default shimming was used in all sequences, no additional shimming was performed. The reference tissue selected for mDixon was ‘skeletal muscle’. From the mDixon reconstructions, the water-only reconstructions were used for comparison to the corresponding SPAIR images.

In T2-weighted images, the mean imaging parameters were as follows for mDixon FSE and SPAIR FSE, respectively: repetition time (TR) 2679 (standard deviation (SD) 53) and 3321 (SD 71) ms, echo time (TE) 63 (SD 0.6) and 60 (SD 0.1) ms, echo train length 16 (SD 0.4) and 14 (SD 0.5), number of signal averages (NSA) 1.4 (SD 0.04) and 1.5 (SD 0.04). In T1-Gd-chelate images with mDixon FSE and SPAIR FSE mean parameters were respectively: TR 676 (SD 16) and 701 (SD 22) ms, TE 13 (SD 5) and 13 (SD 0.6) ms, echo train length 6 (SD 0.1) and 6 (SD 0.1) and NSA 1.1 (SD 0.03) and 1.2 (SD 0.03). Inversion time for the SPAIR sequences was 95 ms.

### Image analysis

mDixon and SPAIR stacks were compared on adjacent monitors using a Sectra viewing system (IDS7 PACS, Linköping, Sweden). Either left or right position of the sequences had been randomized by one of the authors (W. H., not one of the readers). Two radiologists with 33 years (J.B.) and 15 years (C. v R.) of musculoskeletal MRI imaging experience, blinded to the sequence names and clinical information, separately completed a questionnaire comparing eight parameters. Readers based their grade on the whole image stack. Patient order was the same for both readers and study population was read in multiple sessions.

The primary image quality parameter was homogeneity of fat suppression throughout the sequence. Five other parameters included muscle–fat contrast, image noise, random motion artifacts, phase encoded motion artifacts, and edge blurring. Motion artifacts were defined as artifacts due to random motion such as bowel contractions and phase artifacts as ghosting due to repetitive motion, such as breathing. Edge blurring was defined as small lines across the borders of anatomic structures. These six parameters were graded on a five-point scale as follows, 5: perfect image without artifacts, 4: small artifacts at the periphery of the image, 3: prominent artifacts but no interference with the region of interest, 2: prominent artifacts in the region of interest and 1: the region of interest could not be evaluated due to artifacts. The water–fat swap artifact was graded as present or absent. Finally, subjective preference for either technique was recorded when present. Differences between mDixon and SPAIR of two points or more were defined as outliers and were analyzed at a later time by two observers in consensus to determine the reason for this difference.

### Statistical analysis

With a desired power of 0.8, and significance level of 0.05, an anticipated difference of 0.5 points on a scale of 5 in semi-quantitative scoring of imaging parameters and an expected standard deviation of 1.5 points, a minimal study population of 129 patients was needed [15].

Results were collected using the Formdesk questionnaire system (Innovero Software Solutions, Wassenaar, The Netherlands). Statistical analysis was performed in collaboration with the department statistician, using SPSS Statistics version 23 (IBM Corporation, New York, USA). For scan time and inter-reader reliability, the scores were compared with a paired Student’s two-tailed* t* test. To compare mDixon and SPAIR image quality, average scores of the readers were compared with a paired Student’s two-tailed* t* test using a 5% level of significance. A mean difference less than 0.5 points on the five-point scale was considered a non-relevant finding. Six parameters were analyzed with visual grading characteristics (VGC) analysis and area under the curve (AUC). This method, described by Båth [16], was developed to compare image quality on a multiple point scale and uses frequency tables to produce ROC-like curves in order to depict the reader’s preference. Contingency tables were used to detect outlier cases in the primary parameter.

## Results

The scanned body parts are listed in Table 1. The upper trunk included spine, neck, thoracic wall, mediastinum and shoulder. The lower trunk included abdominal wall, retroperitoneum, pelvis, and hips. Extremity scans included elbow, wrist, hand, knee, ankle, and foot. The majority of the scans were performed on knees and pelvis (42 (31%) knees and 24 pelvises (18%) of 135 T2 scans and 64 (50%) knees and 26 pelvises (20%) of 129 T1-Gd scans). A tumor was seen in 81 (60%) of 135 T2 scans and in 68 (53%) of 129 T1-Gd scans. In the cases without a tumor, only post-treatment changes or normal findings were present. The mean acquisition time of the T2-weighted images was 190 (SD 61) seconds for SPAIR and 188 (SD 63) seconds for mDixon (*p* = 0.70, paired *t* test). For T1-weighted post Gd images the acquisition times were 152 (SD 51) seconds for SPAIR and 204 (SD 60) seconds for mDixon (*p* < 0.00, paired * t* test).

**Table 1:** Depicted body parts. Listed are the number of scans. Percentages are listed in brackets

Depicted body parts	T2 (%)	T1-Gd (%)
Upper trunk^a^	29 (22)	22 (17)
Lower trunk^b^	35 (26)	34 (26)
Extremities^c^	71 (53)	73 (57)
Total	135	129

T2 = T2-weighted sequences, T1-Gd = T1-weighted, gadolinium-chelate enhanced sequences

^a^Upper trunk includes spine, neck, mediastinum, thoracic wall and shoulder

^b^Lower trunk includes abdominal wall, retroperitoneum, pelvis and hip

^c^Extremities include elbow, wrist, hand, knee, ankle, and foot

In several parameters (Table 2), inter-reader differences reached significance. However, the difference between average scores was never larger than 0.5 points.

**Table 2:** Inter-reader variability: Mean differences between reader 1 and reader 2 are listed for the spectral attenuated inversion recovery (SPAIR) and modified Dixon (mDixon) images

	T2				T1-Gd			
	mDixon		SPAIR		mDixon		SPAIR	
	Mean difference	*p* value	Mean difference	*p* value	Mean difference	*p* value	Mean difference	*p* value
Homogeneity	− 0.05	0.32	− 0.28	< 0.01	0.11	0.01	0.05	0.50
Contrast	0.08	0.06	− 0.17	0.02	0.15	< 0.01	0.29	< 0.01
Noise	0.21	0.03	0.30	< 0.01	0.02	0.67	0.06	0.41
Motion artifacts	0.30	< 0.01	0.07	0.07	0.14	< 0.01	0.06	0.03
Phase artifacts	− 0.28	< 0.01	− 0.07	0.08	− 0.47	< 0.01	− 0.33	< 0.01
Edge blurring	− 0.07	0.06	− 0.05	0.03	− 0.09	0.04	− 0.02	0.32

Significance was calculated using a two-tailed, paired Student's* t* test. A difference of more than 0.5 points was considered clinically relevant*.* T2 = T2-weighted sequences, T1-Gd = T1-weighted, gadolinium-chelate enhanced sequences

**Fig. 1:**
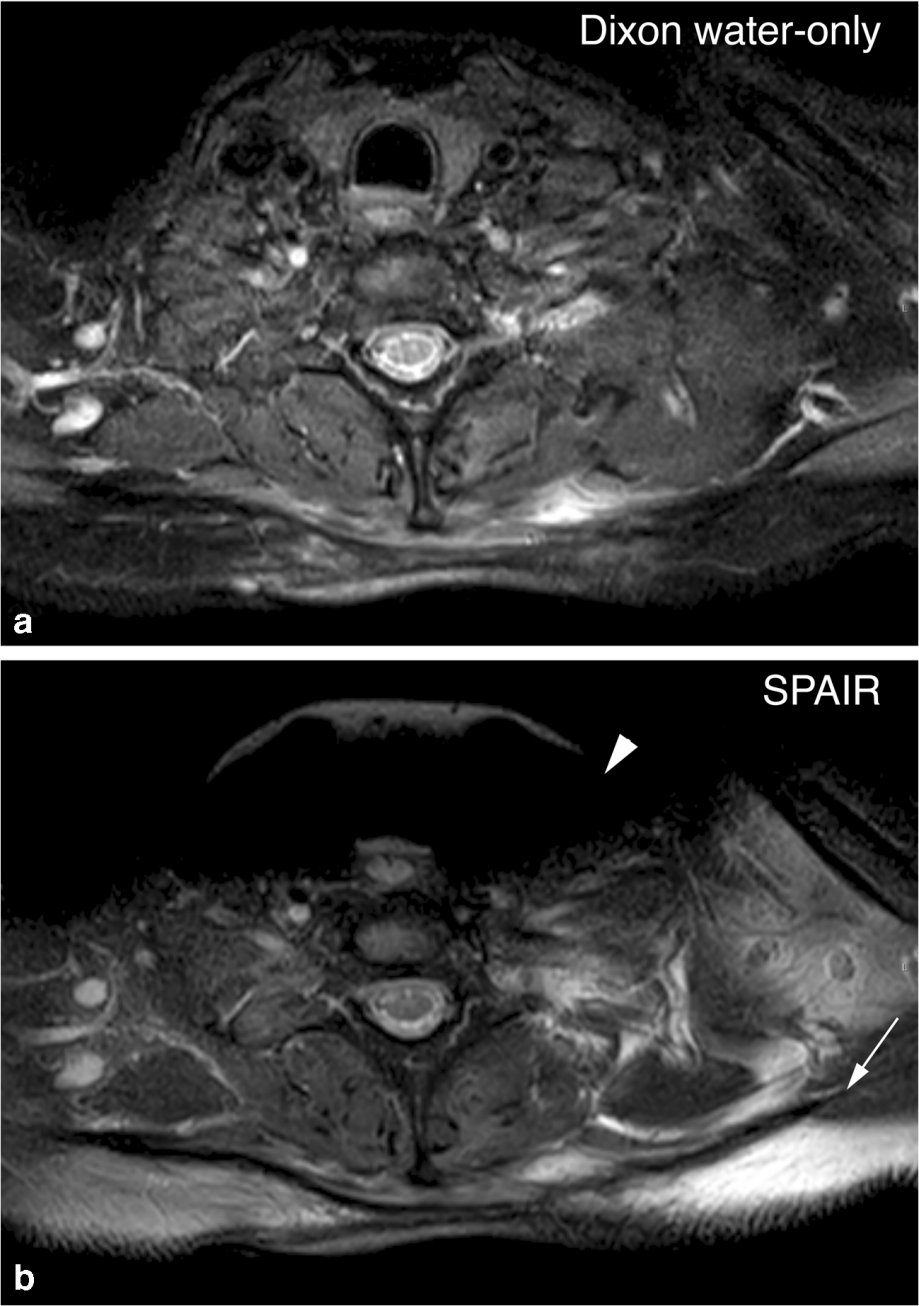
Homogeneity of fat suppression. Axial T2-weighted images of the neck area. In spectral attenuated inversion recovery (SPAIR) imaging (**b**), the signal of fat tissue near the edge of the gantry is not suppressed (*arrow*) due to heterogeneity of the magnetic field. This artifact does not appear in the mDixon water-only image (**a**). Therefore, an area of edema is more conspicuous in the mDixon image. Also, local changes in the magnetic field at the air–tissue interface at the level of the trachea cause large bulk susceptibility artifacts (*arrowhead*) in the SPAIR image. These artifacts are much less prominent in the Dixon water-only image

The scores for mDixon and SPAIR, averaged over the two observers, are listed in Table 3. Average scores of fat suppression homogeneity in the T2-weighted scans were 4.88 (SD 0.35) for mDixon and 4.31 (SD 1.02) for SPAIR (*p* < 0.01). In the T1-Gd-chelate scans mean scores were 4.87 (SD 0.39) for mDixon and 4.21 (SD 1.01) for SPAIR (*p* < 0.01). An example of fat-suppression heterogeneity is shown in Fig. 1.

**Table 3:** Mean scores per parameter: Comparing scores, averaged over the two observers, in spectral attenuated inversion recovery (SPAIR) and modified Dixon (mDixon) images per parameter

	T2				T1-Gd			
	Mean score mDixon	Mean score SPAIR	Difference	*p* value	Mean score mDixon	Mean score SPAIR	Difference	*p* value
Homogeneity	4.88	4.31	0.57	< 0.01	4.87	4.21	0.66	< 0.01
Contrast	4.88	4.66	0.23	< 0.01	4.89	4.70	0.19	< 0.01
Noise	4.31	4.28	0.03	0.44	4.72	4.57	0.15	0.01
Motion artifacts	4.81	4.94	− 0.14	< 0.01	4.93	4.97	− 0.04	0.01
Phase artifacts	4.65	4.90	− 0.25	0.15	4.51	4.54	− 0.03	0.28
Edge blurring	4.85	4.96	− 0.11	< 0.01	4.78	4.98	− 0.21	< 0.01

Significance was calculated using a two-tailed, paired Student's* t* test. A difference of more than 0.5 points was considered clinically relevant. T2 = T2-weighted sequences, T1-Gd = T1-weighted, gadolinium-chelate enhanced sequences

**Fig. 2:**
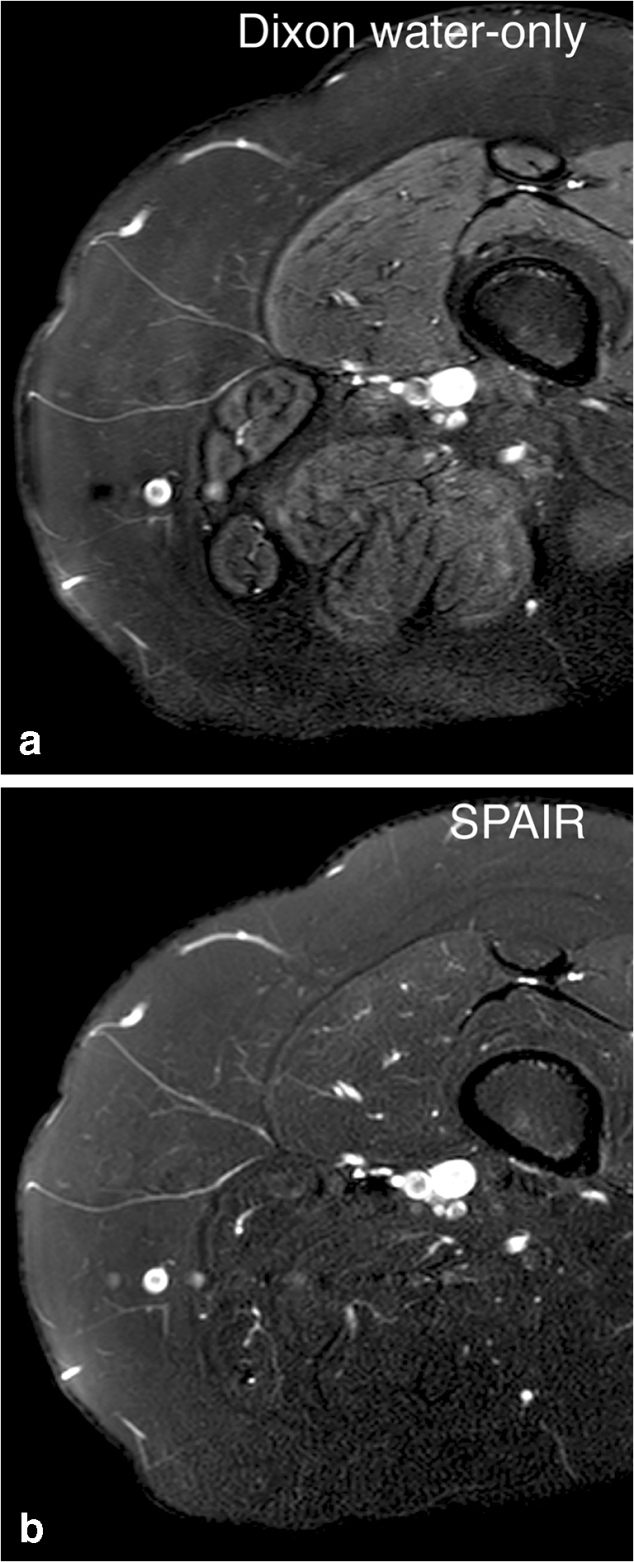
Contrast and noise. Axial T2-weighted images of the right upper leg. Contrast between normal fat and muscle tissue is different for mDixon (**a**) and spectral attenuated inversion recovery (SPAIR) (**b**) images in this case. In the mDixon image, fat exhibits a lower signal intensity compared to muscle because signal from fat is more efficiently eliminated. In the SPAIR image, the signal intensities of both tissues are similar. Both images, scanned in the same location and with the same coil, experience problems with field homogeneity and noise in the periphery (dorsal side of the leg). In the SPAIR image, noise is more prominent and interferes with the visibility of anatomical structures

**Fig. 3:**
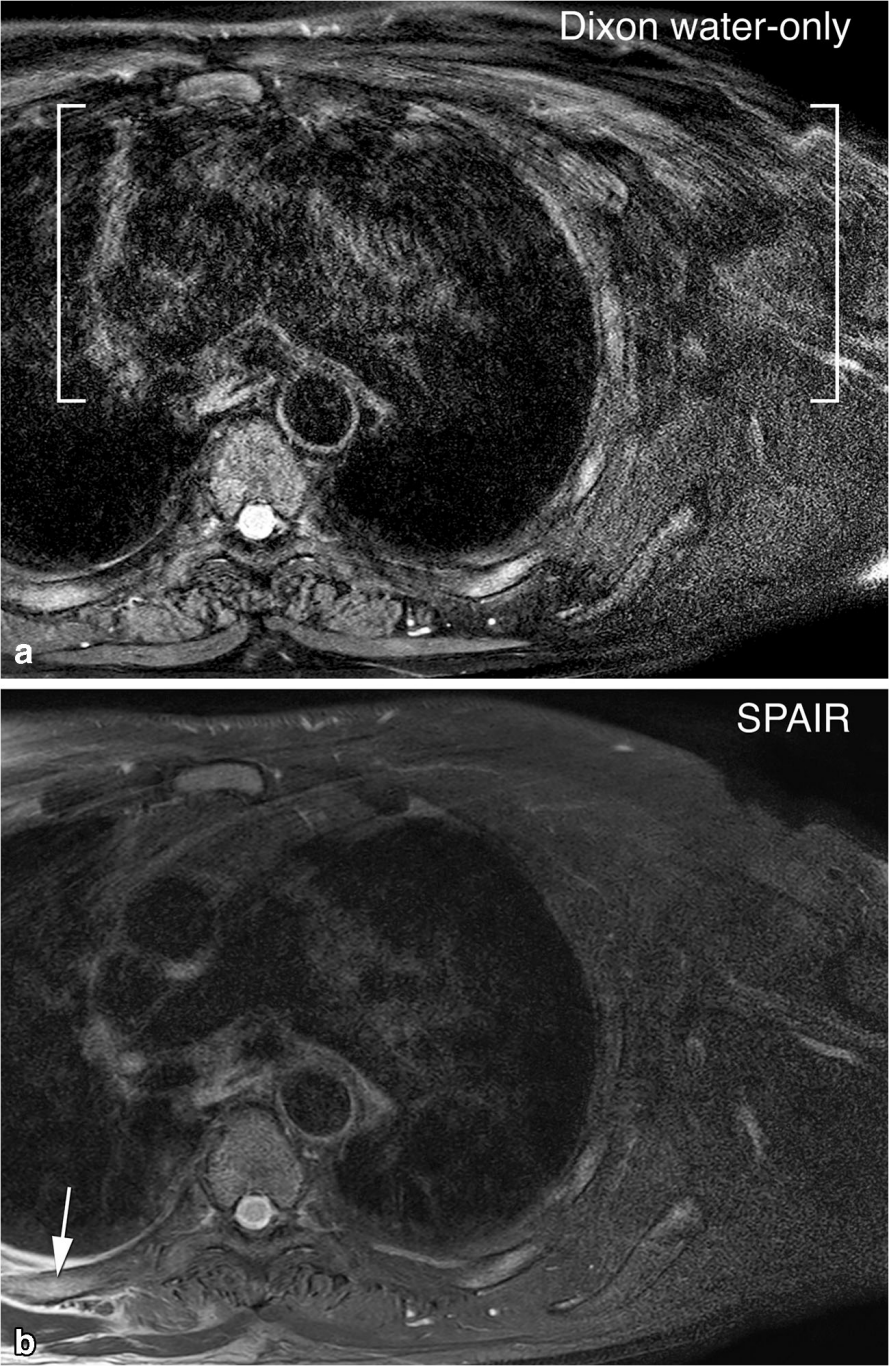
Phase artifacts. Axial T2-weighted images of the thoracic wall. In Dixon water-only imaging (**a**), phase artifacts caused by repetitive cardiac motion are prominent in the phase encoding direction, causing ghosting (indicated by the brackets). The spectral attenuated inversion recovery (SPAIR) image (**b**) is not affected as much. Note, however the incomplete fat suppression in the lower left corner of the SPAIR image (*arrow*)

**Fig. 4:**
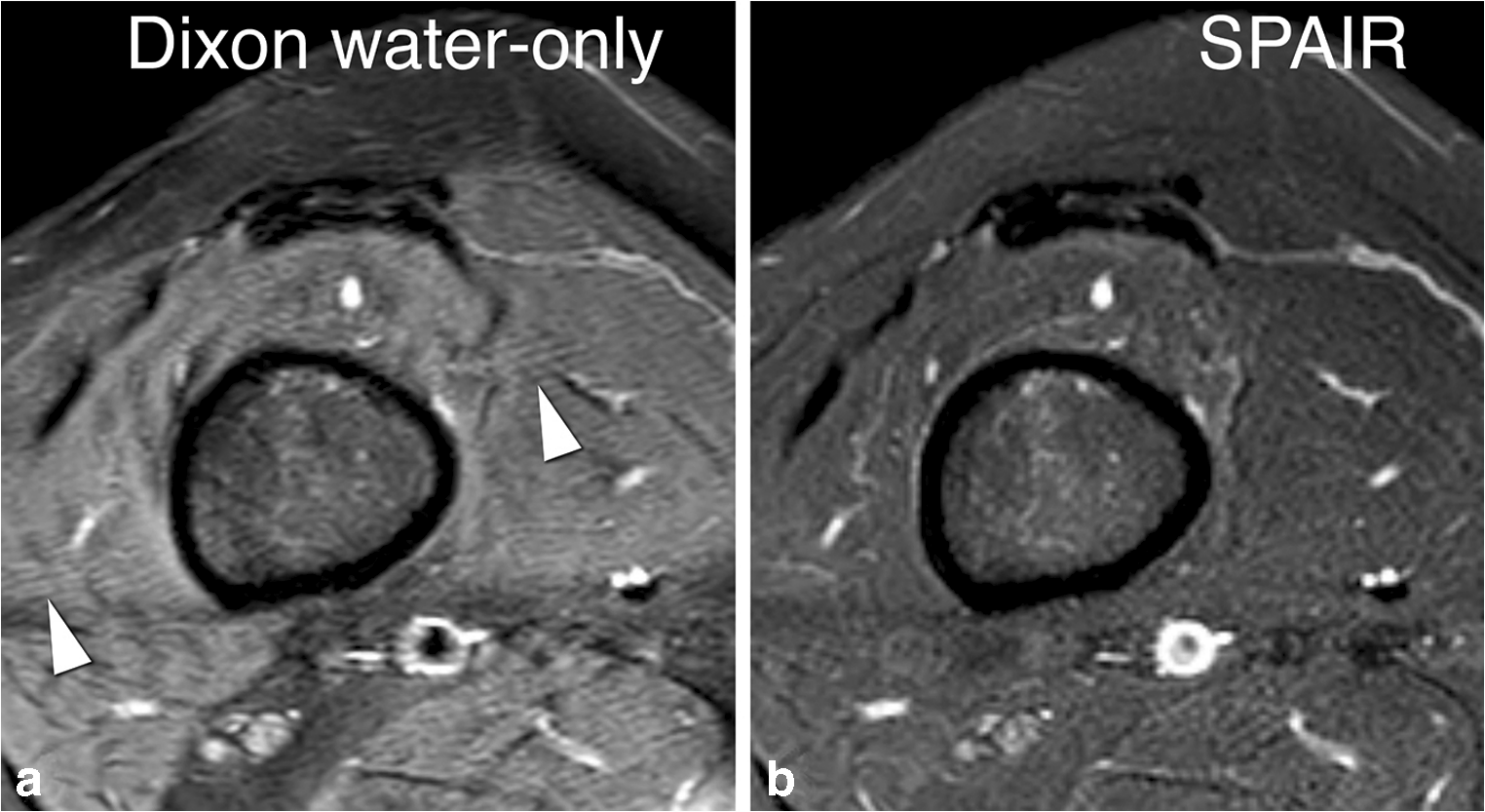
Edge blurring. Axial T2-weighted images of the right upper leg. In the mDixon water-only image (**a**), small parallel lines are visible (*arrowheads*), most prominently at the interface of two tissue types. This artifact is not encountered in the spectral attenuated inversion recovery (SPAIR) image (**b**)

**Fig. 5:**
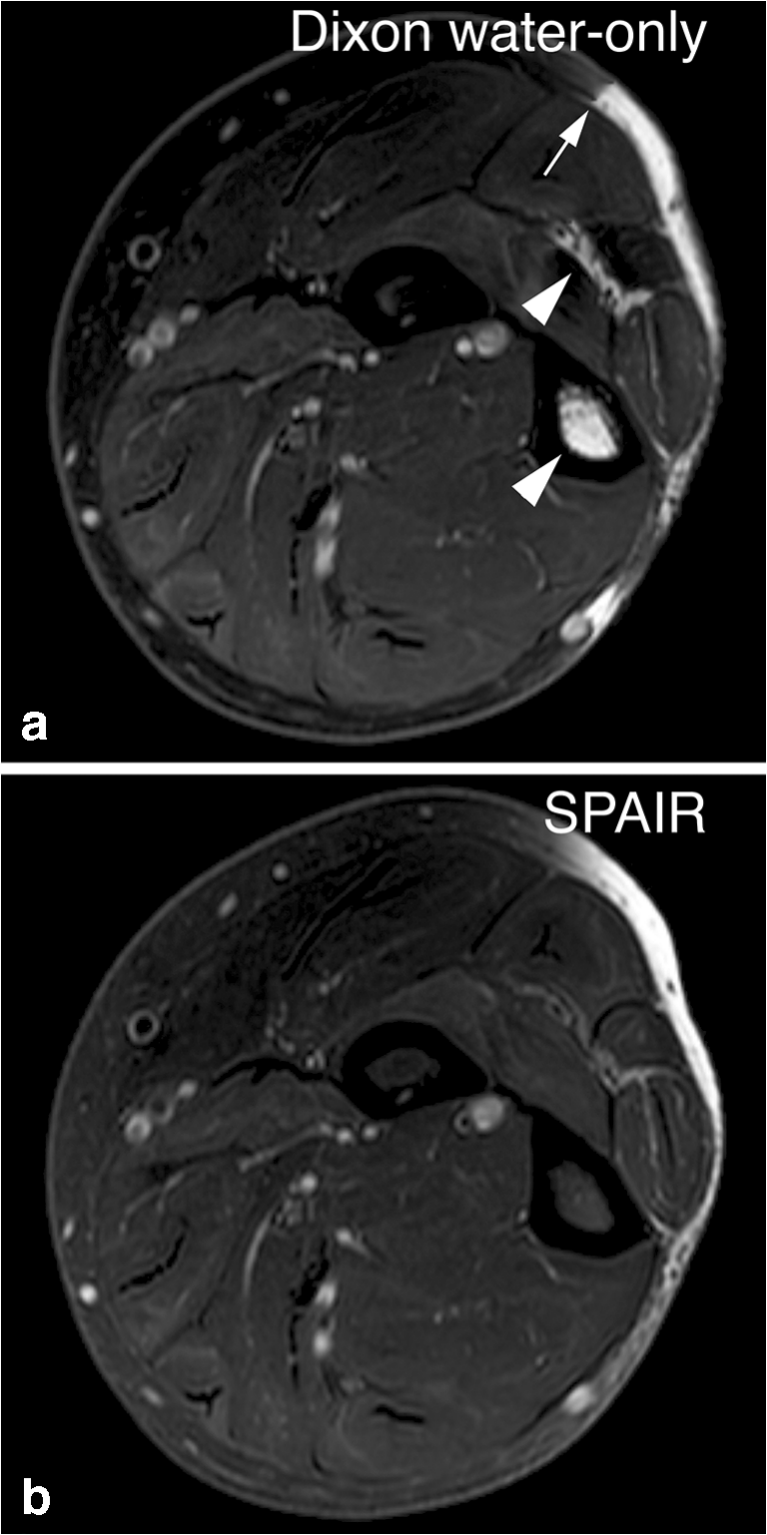
Water–fat swap artifact. Axial T2-weighted images of the left lower arm. Due to the eccentric position of the arm in the gantry, large inhomogeneities were present in the B0 magnetic field. In the spectral attenuated inversion recovery (SPAIR) image (**b**), this causes failure of fat suppression. In the mDixon water-only image (**a**), this causes water–fat swap artifacts: the signal intensity of fat and water are swapped. Thus, high signal is erroneously assigned to the intramedullary cavity, intermuscular tissue (*arrowheads*), and in the subcutaneous fat tissue. Note the sharp demarcation of the water–fat-swap artifact (*arrow*) in the mDixon image

For contrast and noise (Fig. 2), mDixon received slightly higher scores. However, this difference was smaller than 0.5 points and thus not large enough to be relevant according to our predefined criteria. Motion, phase (Fig. 3) and blur artifacts (Fig. 4) were more prominent in mDixon imaging, but again these differences did not reach the 0.5 threshold. Water–fat swap artifacts (Fig. 5) were present in four out of 135 cases (3%) in T2-weighted mDixon images and in two out of 129 cases (2%) in the T1-Gd group.

**Fig. 6:**
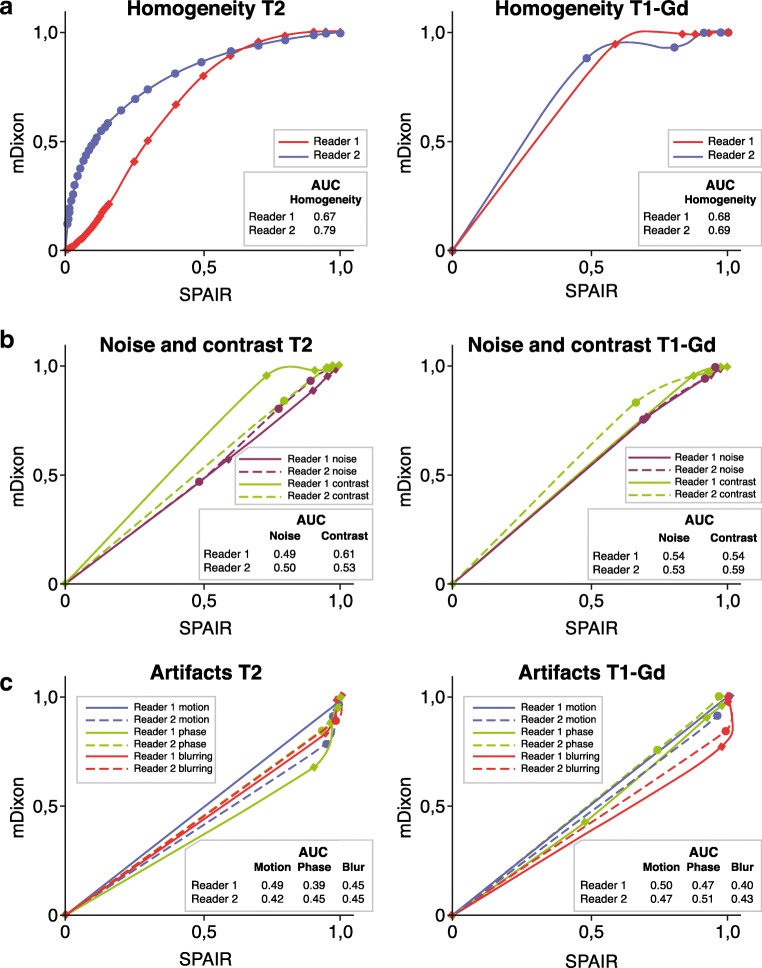
Visual grading characteristics (VGC) curves. The VGC curves of fat-suppression homogeneity for each reader with corresponding area under the curve (AUC) in the* bottom right corner*.** a** VGC curves for reader 1 (*red*) and reader 2 (*blue*).** b** VGC curves for noise (both readers* purple*) and contrast (both readers* green*).** c** VGC curves for motion artifacts (*dark blue*), phase artifacts (*green*) and blurring (*red*)

The visual grading characteristics curves, shown in Fig. 6, demonstrate the degree of preference of each reader for either the mDixon or SPAIR sequence. Both readers showed a preference for mDixon concerning fat-suppression homogeneity, the primary parameter. Areas under the curve were 0.67 for reader 1 and 0.79 for reader 2 in the T2 group and 0.68 for reader 1 and 0.69 for reader 2 in the T1-Gd group. Slight preference for mDixon was found for contrast for reader 1 (AUC 0.61 (T2)) and slight preference for SPAIR in phase artifacts and blur for reader 1 (AUC 0.39 (T2) and 0.40 (T1-Gd)). The other areas under the curve for noise, contrast, and artifacts fell within 0.1 points from 0.5 and were thus categorized as no preference.

For additional outlier analysis of the primary parameter (homogeneity of fat suppression), distinction was made between scores that were higher by 0 or 1 point and scores that were higher by 2 or more points for either mDixon or SPAIR. For overview purposes, all fat-suppression homogeneity scores of both readers are shown in contingency tables in Table 4 (each case was scored by two readers, thus resulting in twice as many scores as cases). The majority of scores fell into the 0–1 point difference group: 228 (84%) in T2 and 222 (86%) in T1-Gd. Of these cases, 95% of sequences contained few artifacts (and scored either four or five points in 218 out of 228 in T2 and 210 out of 222 in T1-Gd).

**Table 4:**
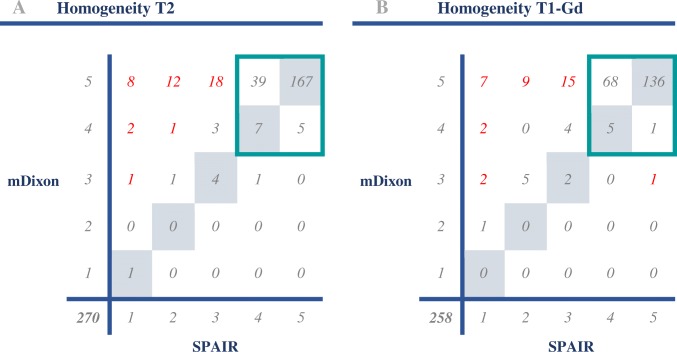
a, b Contingency tables comparing the fat suppression homogeneity scores: All cases were scored by both readers

All scores are listed (270 scores for 135 cases from the T2 group and 258 scores for 129 cases from the T1-Gd group). Scores with two or more points difference between modified Dixon (mDixon) and spectral attenuated inversion recovery (SPAIR) are indicated in red. The scans that received high scores (4 or 5) and differed less than 2 points are marked with a turquoise box. (T2 = T2-weighted images, T1-Gd = T1-weighted gadolinium-chelate enhanced images)

In a smaller amount of scores, the difference was two points or more (from here on referred to as ‘outliers’). These outliers were gathered for further analysis. It should be noted that because each case was scored by both readers, two scores could refer to the same case. The T2-weighted images yielded 42 outlier scores, corresponding to 29 separate cases in favor of mDixon. In the T1-Gd group, outliers were found 35 times, corresponding to 28 separate cases in favor of mDixon and one case in favor of SPAIR.

These outliers were analyzed in order to determine the reason for fat-suppression heterogeneity. Among the outliers in favor of mDixon in the T2-group (29 scans), six SPAIR scans (21%) suffered from bulk susceptibility artifacts (three cervical spines, two shoulders, one foot) and 20 scans (69%) from B_0_ field inhomogeneity problems at the edge of the gantry (seven shoulders, seven elbows, six hips) and six scans (21%) from field inhomogeneity problems at the edge of the coil (one cervical spine, one wrist, three knees, and one foot).

Among the outliers in favor of mDixon in the T1-Gd group (28 scans), five SPAIR scans (18%) suffered from bulk susceptibility artifacts (three cervical spines, one shoulder and one thoracic wall), 16 scans (57%) from field inhomogeneity at the edge of the gantry (five shoulders, five elbows, six hips) and 14 scans (50%) from field inhomogeneity at the edge of the coil (one shoulder, five hips, six knees, one ankle, and one thoracic wall). In this group, mostly sagittal and coronal scans were performed, using the full length of the coil. This is why artifacts at the edge of the coil were more conspicuous than in the T2 group, which were mostly scanned in an axial plane.

The scan of the outlier in favor of SPAIR in T1-Gd was performed in the knee and showed field inhomogeneity problems in the form of water–fat swap artifacts.

The overall subjective preference scores are listed in Table 5 per reader. In more than half of the cases, readers had no preference for either mDixon or SPAIR (55% and 71%). In 19 and 28% of cases, they preferred mDixon and in 9 and 18% of cases they preferred SPAIR.

**Table 5:** Subjective reader preference: In each case, readers were asked to give their subjective preference for modified Dixon (mDixon), spectral attenuated inversion recovery (SPAIR) or neither

	T2 + T1-Gd			
	mDixon	SPAIR	No preference	Total
Reader 1	51 (19%)	25 (9%)	188 (71%)	264
Reader 2	73 (28%)	47 (18%)	144 (55%)	264

The amount of cases in each category are listed, as well as the percentage. T2 = T2-weighted sequences, T1-Gd = T1-weighted, gadolinium-chelate enhanced sequences

## Discussion

The current study shows that the theoretical advantages of mDixon FSE, a fast adaptation of the classic two-point Dixon FSE technique, result in superior quality of T2 fat-suppressed images relative to the SPAIR FSE technique, without disadvantages such as long acquisition times or significant interference by blurring artifacts. Since differences between pulse sequences may vary secondary to specific protocols tailored on clinical indications, we limited our study to a homogeneous population in which a musculoskeletal tumor protocol was clinically indicated.

In the majority of patients, the two observers did not have an overall subjective preference for either mDixon or SPAIR in both T2 and T1 Gd-chelate enhanced sequences, but when looking at the individual image quality parameters, fat suppression with mDixon proved to be significantly more homogeneous than with SPAIR on both T2- and T1-Gd enhanced imaging. Using the predefined threshold of minimally 0.5 point difference on a five-point scale, this was the only parameter of six reaching both a significant difference in the averaged reader scores and a substantial preference with the visual grading characteristics curves for both individual readers. However, the difference on averaged scores was only 0.57 for T2-, and 0.66 for T1-Gd sequences. This means that, overall, in clinical practice the difference between the two sequences is minimal. However, our consensus analysis of outliers showed that mDixon performed strikingly better in a subset of cases with B_0_ field inhomogeneity at the edge of the gantry or coil, and in areas with bulk susceptibility; i.e., around the cervical spine, thoracic wall, shoulder girdle, elbows, and hips.

The secondary parameters showed some significant differences that were considered to be clinically irrelevant because of not reaching the predefined 0.5-point threshold. These differences included better scores for contrast and noise in mDixon and for motion and edge blurring artifacts in SPAIR. We found a recognizable water fat swap in only six out of 264 mDixon exams (2%).

Advantages of Dixon techniques relative to other fat-suppression techniques include reduced dependency on high field strength, high SNR, low specific absorption rate, reduced sensitivity to metal artifact, and insensitivity to B1 inhomogeneity. With two-point mDixon and three-point or four-point Dixon techniques decreased sensitivity to B0 inhomogeneity is achieved [3, 5, 17]. The main disadvantage is the long acquisition time and dependency on reconstruction algorithms with edge blurring due to long echo trains, sensitivity to motion, and phase shifts, and water–fat swap artifacts [3, 4, 18]. In the current study, the use of asymmetrical rather than the classic symmetrical echoes in combination with the reconstruction algorithms of the mDixon technique provide further decreased sensitivity to B_0_ inhomogeneity and eddy currents and decreased echo spacing with shortened acquisition times and less blurring [3].

Dixon fat–water separation can be either gradient echo based or FSE based. The mDixon sequence can also be applied to both, using a bipolar gradient readout for gradient echo and a multi-repetition spin echo sequence with flexible TE values for FSE [6]. Because we aimed to evaluate the routinely used FSE techniques in MSK tumor imaging, we did not use gradient echo mDixon techniques.

Mentioned in the European Society of Musculoskeletal Radiology guidelines for soft tissue tumor imaging [19] and practiced in many tumor centers is the use of T2-weighted images both with and without fat saturation. The possibility to save time by using both the water-only and in-phase reconstructions from the same Dixon acquisition to replace these sequences was beyond the scope of the current study. Interestingly, the use of the other Dixon reconstructions (in-phase, out-of-phase and fat-only reconstructions) in MSK imaging are being studied by other groups, including the possibilities for fat quantification [20, 21] and might contribute to saving time by replacement of other sequences. In the future, mDixon may allow other innovations such as real-time change of water–fat contributions to an image at the work station.

There are several limitations in our study. Firstly, although we aimed to use the same acquisition times for both mDixon FSE and SPAIR FSE, the T1-Gd mDixon sequence was on average 50 s longer than SPAIR. Unfortunately, the technicians took liberty to deviate from the protocol in the T1-Gd-chelate sequences in an attempt to improve image quality, but there was no difference in acquisition times between the T2-weighted SPAIR and mDixon sequences. Secondly, although readers were blinded to protocol name, it is likely that they could deduce which sequence was the mDixon sequence due to inherent differences in contrast and based on the type of artifacts. Thirdly, the image quality was only studied at 1.5 T because we aimed at studying a homogeneous data set and we schedule more musculoskeletal oncology patients on our 1.5 T than on 3-T scanners. Potentially, differences in image quality will be larger when using a 3-T system because magnet heterogeneity increases with field strength. Image quality is difficult to quantify and depends on personal preference and display settings. Finally, there were significant differences between reader scores. However, these differences were small and considered irrelevant.

Concluding, in a musculoskeletal oncology population, mDixon FSE at 1.5 T allows time-effective creation of T2-weighted images with superior elimination of fat relative to SPAIR FSE images and without disadvantages. Especially in areas of field inhomogeneity mDixon is preferred. When motion is an issue, SPAIR is preferred. In our tumor protocols, we replaced T2-SPAIR FSE by mDixon FSE.
